# Activated protein C ameliorates impaired renal microvascular oxygenation and sodium reabsorption in endotoxemic rats

**DOI:** 10.1186/2197-425X-1-5

**Published:** 2013-10-29

**Authors:** Emre Almac, Tanja Johannes, Rick Bezemer, Egbert G Mik, Klaus E Unertl, AB Johan Groeneveld, Can Ince

**Affiliations:** Department of Translational Physiology, Academic Medical Center, University of Amsterdam, Meibergdreef 9, 1105 AZ Amsterdam, The Netherlands; Department of Anesthesiology and Intensive Care, St. Antonius Hospital, Nieuwegein, The Netherlands; Department of Anesthesiology, Erasmus MC University Medical Center, Rotterdam, The Netherlands; Department of Anesthesiology and Intensive Care Medicine, University Hospital Tübingen, Tübingen, Germany; Department of Intensive Care, Erasmus MC University Medical Center, Rotterdam, 3015 GE The Netherlands

**Keywords:** Endotoxemia, APC, Activated protein C, AKI, Microcirculation

## Abstract

**Introduction:**

We aimed to test whether continuous recombinant human activated protein C (APC) administration would be able to protect renal oxygenation and function during endotoxemia in order to provide more insight into the role of coagulation and inflammation in the development of septic acute kidney injury.

**Methods:**

In anesthetized, mechanically ventilated Wistar rats, endotoxemia was induced by lipopolysaccharide administration (10 mg/kg i.v. over 30 min). One hour later, the rats received fluid resuscitation with 0 (LPS + FR group; *n* = 8), 10 (APC10 group; *n* = 8), or 100 (APC100 group; *n* = 8) μg/kg/h APC for 2 h. Renal microvascular oxygenation in the cortex and medulla were measured using phosphorimetry, and renal creatinine clearance rate and sodium reabsorption were measured as indicators of renal function. Statistical significance of differences between groups was tested using two-way ANOVA with Bonferroni *post hoc* tests.

**Results:**

APC did not have notable effects on systemic and renal hemodynamic and oxygenation variables or creatinine clearance. The changes in renal microvascular oxygenation in both the cortex (*r* = 0.66; *p* < 0.001) and medulla (*r* = 0.80; *p* < 0.001) were correlated to renal sodium reabsorption_._

**Conclusion:**

Renal sodium reabsorption is closely correlated to renal microvascular oxygenation during endotoxemia. In this study, fluid resuscitation and APC supplementation were not significantly effective in protecting renal microvascular oxygenation and renal function. The specific mechanisms responsible for these effects of APC warrant further study.

**Electronic supplementary material:**

The online version of this article (doi:10.1186/2197-425X-1-5) contains supplementary material, which is available to authorized users.

## Introduction

The prevalence of acute kidney injury (AKI) in septic patients is high, approximately 20% to 50%, and the AKI-related mortality rate is 75% in septic shock compared to 45% without sepsis [1, 2]. The pathogenesis and pathophysiology of sepsis-induced AKI is highly complex, and current treatment strategies are mainly supportive rather than curative [3]. There is a growing body of evidence that microcirculatory dysfunction accompanied by tissue dysoxia might play a key role in the development of septic organ damage [4, 5]. Excessive release of pro-inflammatory mediators and disturbances in the coagulation system are believed to be involved in the pathogenesis of sepsis; both leading to microcirculatory dysfunction and consequent organ failure [6, 7].

Activated protein C (APC) is an important endogenous protein that modulates coagulation and inflammation by promoting fibrinolysis and inhibiting thrombosis and inflammation [8–10]. Different experimental and clinical studies could demonstrate that the administration of APC improved outcome of severe sepsis [11–15]. With respect to the protective effects of APC on the kidney during sepsis, especially the group of Gupta et al. has identified specific mechanisms of action of APC in rat models of experimentally induced septic AKI [16–19]. Another group, furthermore, showed that APC reduced ischemia/reperfusion (*I*/*R*)-induced renal injury in rats [20].

Besides its potentially beneficial effects, there are serious concerns regarding the safety and efficacy of APC treatment in critically ill septic patients [21]. These concerns led to discontinuation of all ongoing clinical trials using APC for treatment of severe sepsis. APC is no longer suggested for the treatment of severe sepsis or septic shock. Despite these developments, investigating the effects of APC in experimental studies may provide more insights into the development of septic AKI.

The above mentioned studies have provided key insights into the beneficial effects of APC in sepsis; however, none of these studies have investigated the effects of APC on renal oxygenation and function in terms of tubular sodium reabsorption. In the present study, we tested whether continuous recombinant human APC administration would be able to protect renal oxygenation and function during the acute phase of endotoxemia and fluid resuscitation.

## Materials and methods

All experiments in this study were approved by the institutional Animal Experimentation Committee of the Academic Medical Center of the University of Amsterdam (institutional protocol number: DFL 100404). Care and handling of the animals were in accordance with the guidelines for Institutional and Animal Care and Use Committees. The experiments were performed on 32 Wistar male rats (Harlan Laboratories, Inc., Boxmeer, The Netherlands) with mean ± SD body weight of 318 ± 15 g.

### Surgical preparation

The rats were anesthetized with an intraperitoneal injection of a mixture of 100 mg/kg ketamine (Nimatek®, Eurovet, Bladel, The Netherlands), 0.5 mg/kg medetomidine (Domitor, Pfizer Inc., New York, NY, USA), and 0.05 mg/kg atropine sulfate (Centrafarm Pharmaceuticals B.V., Etten-Leur, The Netherlands). After tracheotomy, the animals were mechanically ventilated with a FiO_2_ of 0.4. Body temperature was maintained at 37°C ± 0.5°C during the entire experiment by external warming. The ventilator settings were adjusted to maintain end-tidal PCO_2_ between 30 and 35 mmHg, and arterial PCO_2_ between 35 and 40 mmHg.

The vessels were cannulated with polyethylene catheters (outer diameter = 0.9 mm; B. Braun Melsungen AG, Melsungen, Germany) for drug and fluid administration and hemodynamic monitoring. A catheter in the right carotid artery was connected to a pressure transducer to monitor the mean arterial blood pressure (MAP) and heart rate. The right femoral artery was cannulated for blood sampling. The right femoral vein was cannulated for continuous infusion of Ringer’s lactate (15 mL/kg/h; Baxter B.V., Utrecht, The Netherlands) and ketamine (50 mg/kg/h; Nimatek®; Eurovet).

The left kidney was exposed, decapsulated, and immobilized in a Lucite kidney cup (K. Effenberger, Pfaffingen, Germany) via a 4-cm incision in the left flank. The renal vessels were carefully separated under preservation of nerves and adrenal gland. A perivascular ultrasonic transient time flow probe was placed around the left renal artery (type 0.7 RB; Transonic Systems Inc., Ithaca, NY, USA) and connected to a flow meter (T206; Transonic Systems Inc.) to continuously measure the renal blood flow (RBF). An estimation of the renal vascular resistance (RVR) was made as RVR (dynes s^−1^ cm^−5^) = (MAP / RBF). The left ureter was isolated, ligated, and cannulated with a polyethylene catheter for urine collection.

After the surgical protocol (approximately 60 min), one optical fiber was placed 1 mm above the decapsulated kidney and another optical fiber 1 mm above the renal vein to measure oxygenation in the renal microvasculature and renal vein, respectively, using phosphorimetry [22–24]. A small piece of aluminum foil was placed on the dorsal site of the renal vein to prevent contribution of underlying tissue to the phosphorescence signal in the venous oxygenation measurement. Oxyphor G2 (a two-layer glutamate dendrimer of tetra-(4-carboxy-phenyl) benzoporphyrin; Oxygen Enterprises Ltd., Philadelphia, PA, USA) was subsequently infused (6 mg/kg IV over 5 min) followed by a 30-min stabilization period. A short description of phosphorimetry is given below, and a more detailed description of the technology has been provided elsewhere [22, 23].

### Experimental protocol

After baseline measurements were performed 30 min after Oxyphor G2 infusion, endotoxemic shock was induced in three groups of rats (*n* = 8/group) by a bolus of lipopolysaccharide (LPS, 10 mg/kg, serotype 0127:B8, Sigma-Aldrich, Zwijndrecht, The Netherlands). One hour after the LPS bolus, fluid resuscitation (5 mL/kg followed by 5 mL/kg/h; Voluven®, 6% HES 130/0.4; Fresenius Kabi, Schelle, Belgium) was started and continued for 2 h. In addition to the fluid resuscitation, one group received 10 μg/kg/h APC and one group received 100 μg/kg/h APC (recombinant human activated protein C; Drotrecogin Alpha, Xigris®, Eli Lilly and Company, Indianapolis, IN, USA). A fourth group of rats (*n* = 8) did not receive LPS or fluid resuscitation and served as a sham-operated time control group. All the rats, except for those in the time control group, received the same fluid volume. The experiments were terminated by infusion of 1 mL of 3 M potassium chloride (KCl), after which the kidneys were removed and weighted.

### Blood variables

Arterial blood samples (0.5 mL) were taken from the femoral artery at the following time points: (1) baseline; (2) 1 h after the LPS bolus, before the start of fluid resuscitation; and (3) after 2 h of fluid resuscitation. The blood samples were replaced by the same volume of Voluven®. The samples were analyzed for blood gas values (ABL505 blood gas analyzer; Radiometer Medical ApS, Copenhagen, Denmark), hemoglobin concentration, and hemoglobin oxygen saturation (OSM3; Radiometer Medical ApS). Additionally, plasma creatinine concentrations were determined in all the samples.

### Renal microvascular and venous oxygenation

Microvascular oxygen tension in the renal cortex (CμPO_2_), outer medulla (MμPO_2_), and renal venous oxygen tension (P_rv_O_2_) were measured by oxygen-dependent quenching of phosphorescence lifetimes of the systemically infused albumin-targeted (and therefore circulation-confined) phosphorescent dye Oxyphor G2 [24]. Oxyphor G2 (a two-layer glutamate dendrimer of tetra-(4-carboxy-phenyl) benzoporphyrin) has two excitation peaks (*λ*_excitation1_ = 440 nm, *λ*_excitation2_ = 632 nm) and one emission peak (*λ*_emission_ = 800 nm). These optical properties allow (near) simultaneous lifetime measurements in microcirculation of the kidney cortex and the outer medulla due to different optical penetration depths of the excitation light [25]. For the measurement of renal venous PO_2_ (P_rv_O_2_), a mono-wavelength phosphorimeter was used [26]. Oxygen measurements based on phosphorescence lifetime techniques rely on the principle that phosphorescence can be quenched by energy transfer to oxygen, resulting in shortening of the phosphorescence lifetime. A linear relationship between reciprocal phosphorescence lifetime and oxygen tension (given by the Stern-Volmer relation) allows quantitative measurement of PO_2_ [27].

### Renal oxygen delivery and consumption

Arterial oxygen content (AOC) was calculated by (1.31 × hemoglobin × S_a_O_2_) + (0.003 × P_a_O_2_), where S_a_O_2_ is the arterial oxygen saturation, and P_a_O_2_ is the arterial partial pressure of oxygen. Renal venous oxygen content (RVOC) was calculated as (1.31 × hemoglobin × S_rv_O_2_) + (0.003 × P_rv_O_2_), where S_rv_O_2_ is the venous oxygen saturation, and P_rv_O_2_ is the renal vein partial pressure of oxygen (measured using phosphorimetry). Renal oxygen delivery per gram of renal tissue was calculated as DO_2_ (mL/min/g) = RBF × AOC. Renal oxygen consumption per gram of renal tissue was calculated as VO_2_ (mL/min/g) = RBF × (AOC − RVOC). The renal oxygen extraction ratio was calculated as O_2_ER (%) = VO_2_/DO_2_ × 100.

### Renal function

For the analysis of urine volume, creatinine concentration, and sodium (Na^+^) concentration at the end of the protocol, urine samples from the left ureter were collected for 10 min. Creatinine clearance rate (CL_crea_) per gram of renal tissue was calculated with standard formula: CL_crea_ (mL/min/g) = (*U* × *V*) / *P*, where *U* is the urine creatinine concentration, *V* is the urine volume per unit time, and *P* is the plasma creatinine concentration. Renal sodium reabsorption (T_Na+_, (mmol/min)) was calculated as T_Na+_ = (*P*_Na+_ × CCR) − (*U*_Na+_ × *V*), where *U*_Na+_ is the urine sodium concentration, and *P*_Na+_ is the plasma sodium concentration. The renal oxygen consumption efficiency for sodium transport (VO_2_/T_Na+_) was assessed as the ratio of the renal VO_2_ over the total amount of sodium reabsorbed (T_Na+_, (mmol/min)).

### Data analysis

Statistical analysis was performed using GraphPad Prism version 5.0 for Windows (GraphPad Software Inc., San Diego, CA, USA). Data are presented as median (25% to 75% percentiles). The statistical significance of differences between groups was tested using two-way ANOVA with Bonferroni *post hoc* tests. *P* values < 0.05 were considered significant.

## Results

**Figure 1:**
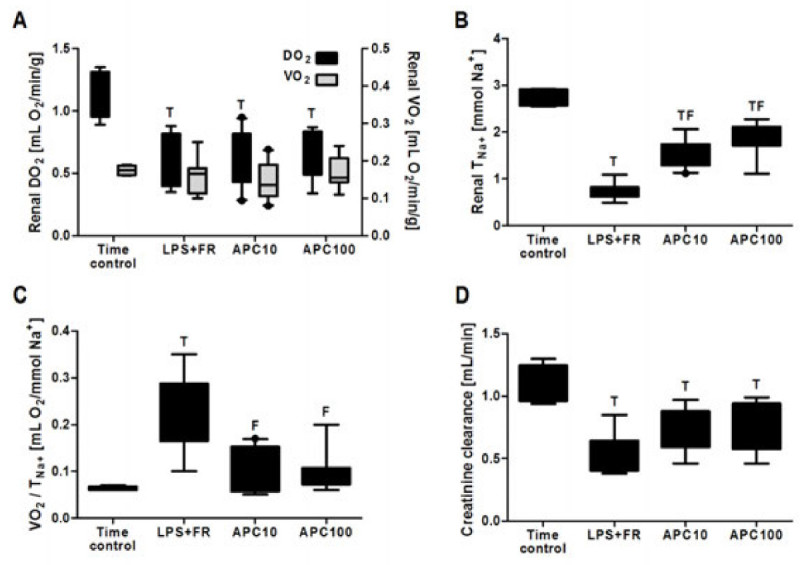
**DO**_**2**_ **,** **VO**_**2**_ **,** **T**_**Na+**_ **,** **VO**_**2**_ **/** **T**_**Na+**_ **,** **and creatinine clearance rate at the end of protocol.** Data are presented as Whisker boxes and range. ^T^ *p* < 0.05 vs time control; ^F^ *p* < 0.05 vs LPS + FR. **(A)** Renal oxygen delivery and consumption, **(B)** renal sodium reabsorption, **(C)** renal oxygen handling, and **(D)** renal creatinine clearance rate.

Table 1 shows the systemic and renal hemodynamic variables: mean arterial pressure (MAP), renal blood flow (RBF), renal vascular resistance (RVR), arterial hemoglobin level (Hb), renal oxygen delivery (DO_2_), renal oxygen consumption (VO_2_), and microvascular oxygen tensions in the renal cortex (CμPO_2_) and medulla (MμPO_2_) at baseline (BL); 1 h after the LPS bolus, before the start of fluid resuscitation (LPS); and after 2 h of fluid resuscitation (FR). Figure 1 shows the renal DO_2_ and VO_2_ and T_Na+_, renal oxygen handling efficacy (VO_2_/T_Na+_), and creatinine clearance rate at the end of the protocol. No differences in any of these variables between groups were present at baseline.

**Table 1:** **Systemic and renal hemodynamic variables in the renal cortex and medulla at three sampling points**

	BL (***t***= 0 min)		LPS (***t***= 60 min)			FR (***t***= 180 min)		
MAP (mmHg)								
Time control	99	(97–103)	99	(94–99)		90	(85–94)	
LPS + FR	102	(96–109)	77	(73–93)	^**T**^	68	(44–79)	^**T**^
APC10	100	(99–102)	76	(69–84)	^**T**^	72	(59–78)	^**T**^
APC100	102	(102–103)	78	(76–92)	^**T**^	81	(70–89)	^**T**^
RBF (mL/min)								
Time control	6.2	(5.8-6.3)	5.9	(4.7-6.1)		5.1	(4.7-5.8)	
LPS + FR	6.8	(6.0-6.8)	3.1	(3.1-3.3)	^**T**^	4.6	(2.2-5.1)	^**T**^
APC10	6.6	(5.0-7.1)	3.2	(1.7-4.5)	^**T**^	4.1	(2.8-5.1)	^**T**^
APC100	6.0	(5.9-6.8)	3.2	(2.3-4.2)	^**T**^	4.2	(2.8-4.7)	^**T**^
RVR (dyn s^−1^ cm^−5^)								
Time control	1,319	(1,251-1,373)	1,347	(1,301-1,579)		1,332	(1,280-1,558)	
LPS + FR	1,276	(1,183-1,407)	1,901	(1,620-2,673)	^**T**^	1,311	(828–2,314)	
APC10	1,267	(1,124-1,635)	2,086	(1,353-3,222)	^**T**^	1,371	(938–2,038)	
APC100	1,331	(1,203-1,407)	2,024	(1,761-2,743)	^**T**^	1,636	(1,383-1,864)	
Hb (g/dL)								
Time control	0.19	(0.18-0.20)	0.18	(0.17-0.20)		0.18	(0.15-0.19)	
LPS + FR	0.19	(0.17-0.20)	0.17	(0.17-0.18)		0.13	(0.12-0.14)	^**T**^
APC10	0.19	(0.18-0.20)	0.19	(0.17-0.20)		0.13	(0.12-0.14)	^**T**^
APC100	0.19	(0.18-0.19)	0.18	(0.17-0.19)		0.13	(0.11-0.14)	^**T**^
DO_2_ (mL O_2_/min/g)								
Time control	0.90	(0.82-0.95)	0.80	(0.60-0.93)		0.69	(0.56-0.78)	
LPS + FR	0.87	(0.77-0.96)	0.39	(0.37-0.43)	^**T**^	0.46	(0.22-0.48)	^**T**^
APC10	0.89	(0.81-0.99)	0.41	(0.26-0.63)	^**T**^	0.40	(0.25-0.52)	^**T**^
APC100	0.85	(0.80-0.97)	0.45	(0.28-0.61)	^**T**^	0.37	(0.29-0.49)	^**T**^
VO_2_ (mL O_2_/min/g)								
Time control	0.16	(0.12-0.20)	0.18	(0.08-0.26)		0.18	(0.16-0.19)	
LPS + FR	0.13	(0.12-0.17)	0.11	(0.08-0.15)		0.17	(0.11-0.18)	
APC10	0.18	(0.12-0.19)	0.13	(0.07-0.16)		0.14	(0.11-0.20)	
APC100	0.17	(0.13-0.24)	0.12	(0.10-0.17)		0.16	(0.14-0.21)	
CμPO_2_ (mmHg)								
Time control	86	(82–87)	85	(78–92)		71	(66–79)	
LPS + FR	83	(77–87)	69	(65–77)	^**T**^	49	(43–51)	^**T**^
APC10	82	(81–87)	75	(65–83)	^**T**^	56	(49–59)	^**T**^
APC100	80	(75–88)	67	(64–75)	^**T**^	68	(54–71)	
MμPO_2_ (mmHg)								
Time control	65	(59–66)	62	(57–67)		58	(53–60)	
LPS + FR	55	(51–66)	55	(50–57)	^**T**^	40	(35–44)	^**T**^
APC10	62	(55–64)	53	(47–60)	^**T**^	45	(39–49)	^**T**^
APC100	59	(55–69)	55	(49–59)	^**T**^	55	(49–58)	

Data are presented as median (25% to 75% percentiles). MAP, mean arterial pressure; RBF, renal blood flow; RVR, renal vascular resistance; Hb, arterial hemoglobin level; DO_2_, renal oxygen delivery; VO_2_, renal oxygen consumption; CμPO_2_, microvascular oxygen tension in the renal cortex; microvascular oxygen tension in the medulla (MμPO_2_); BL, baseline (BL); LPS, lipopolysaccharide; FR, fluid resuscitation. ^T^ *p* < 0.05 vs time control.

### Systemic and renal hemodynamic variables

The bolus of LPS (10 mg/kg) induced a significant drop in MAP and RBF, and a rise in RVR. One hour after LPS administration, all the rats received the same amount of Voluven® during the resuscitation protocol, i.e., a bolus of 5 mL/kg followed by 5 mL/kg/h for 2 h. Fluid resuscitation could not improve MAP, RBF, and RVR back to baseline level. No additional effects of APC supplementation (10 or 100 μg/kg/h) on systemic and renal hemodynamic variables were observed.

### Renal oxygenation variables

In line with RBF, renal DO_2_ decreased after the LPS bolus in all groups. Fluid resuscitation led to a reduction in the arterial hemoglobin concentration, and therefore, in contrast to RBF, renal DO_2_ did not improve due to the hemodilution. At the end of the protocol, despite differences in MAP and RBF between the groups, there were no significant differences in DO_2_. The reduced renal DO_2_ was also reflected by the reduced microvascular oxygenation in the renal cortex and medulla in all groups. In the APC100 group, the cortical and medullar PO_2_ was slightly higher than those in other experimental groups; however, this difference was not statistically significant. Even though renal DO_2_ was decreased after LPS and fluid administration, renal VO_2_ was maintained throughout the entire protocol. No additional effects of APC supplementation on renal DO_2_ and VO_2_ were observed.

### Renal function parameters

**Figure 2:**
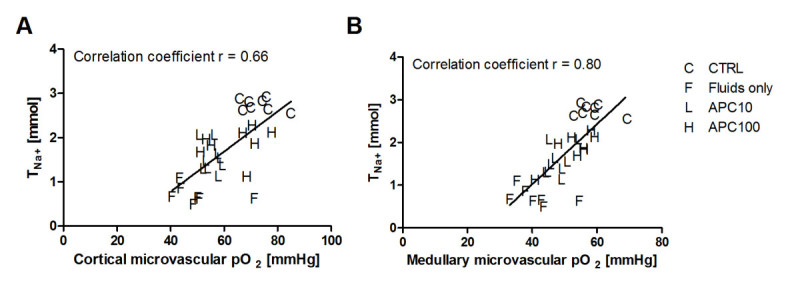
**Renal sodium reabsorption** **(T**_**Na+**_ **)** **versus the microvascular oxygenation.** In the renal cortex **(A)** and medulla **(B)** at the end of the protocol in all groups.

At the end of the protocol, creatinine clearance rate and sodium reabsorption had decreased in all endotoxemic groups. Supplementation with APC improved both parameters, albeit not to baseline level. Renal oxygen handling efficacy, as expressed as the amount of oxygen consumed by the kidney per sodium reabsorbed (VO_2_/T_Na+_), was increased fourfold in the endotoxemic group receiving only fluids without APC and less than two-old in the groups receiving APC. Figure 2 shows that renal sodium reabsorption was closely correlated to renal microvascular oxygenation during endotoxemia and resuscitation, and APC supplementation partially protects both renal parameters.

## Discussion

It has been shown in rat models of experimentally induced sepsis that APC has protective effects on the kidney [16–19]. These studies have provided important insights into both the pathophysiology of septic AKI and the potential role of APC in its prevention and/or treatment. In the present study, we aimed to test whether continuous recombinant human APC administration would be able to protect renal oxygenation and function during the acute phase of lipopolysaccharide-induced endotoxemia and fluid resuscitation. Although APC has been withdrawn from the market, investigating its effects in studies like this is still very relevant as it might provide more insight into the development of septic AKI.

In our model, endotoxemia and fluid resuscitation led to progressive renal vasoconstriction (increased RVR and decreased RBF) and a decrease in renal DO_2_ and microvascular oxygenation, a fall in glomerular filtration rate (decreased creatinine clearance), and a fourfold rise in the amount of oxygen consumed by the kidney per sodium reabsorbed (VO_2_/T_Na+_). Our main findings regarding the effects of APC were that APC did not have significant effects on the systemic and renal hemodynamic and oxygenation variables or creatinine clearance. In the fluid resuscitation group, MAP was slightly lower than those in the other experimental groups, however not significant. Despite lower MAP values, RBF was higher than those in the APC10 and APC100 groups. There were no statistical differences in DO_2_ between groups at the end of the protocol. Particularly, in the APC100 group, both renal cortical and medullary microvascular oxygenation were better preserved than in fluid resuscitation alone. However, this difference was not statistically significant. In addition, sodium reabsorption and oxygen consumption per sodium reabsorbed (VO_2_/T_Na+_) were preserved in the APC10 and APC100 groups as compared to fluid resuscitation alone. Renal sodium reabsorption was closely correlated to renal microvascular oxygenation during endotoxemia and resuscitation, suggesting a better renal oxygen handling and less renal damage. Nevertheless, there was no assessment of cellular hypoxia or damage to confirm the suggestion above.

Although the mechanisms underlying acute renal failure are not completely defined, in the early stage of sepsis, impairment of the renal microcirculation is believed to be a key complication potentially leading to renal failure [25, 28–30]. In addition to an imbalance between physiological vasoactive compounds, it has been suggested that hypoxic microvascular areas might arise in the renal cortex in untreated endotoxemia [5]. These hypoxic areas are considered to reflect shunting of weak microcirculatory units [31, 32]. In the present study, 100 μg/kg APC-treated rats had less reduction of microvascular oxygenation than that observed in the other groups. As mentioned above, this was not statistically significant due to small-sized groups. Also, MAP was slightly higher in APC-treated rats, compared to that with fluid resuscitation alone. It might be suggested that the changes in the renal microvascular oxygenation are related to those in systemic blood pressure. However, RBF and DO2 were not influenced by the mild changes in systemic blood pressure.

Furthermore, APC treatment did not affect renal DO_2_ or VO_2_. This could also be explained by reduced microvascular shunting in the APC100 group: when blood is shunted from the microcirculation, the remaining microcirculatory blood would deoxygenate more rapidly, while venous oxygenation would be maintained as this is mixed microcirculatory blood and shunted blood from the arterial side. It has been shown that serine protease protein C plays an important role in controlling thrombosis and inflammation and that it exhibits cytoprotective properties [26]. There is evidence that in septic patients, a reduced plasma level of protein C is prognostic for clinical outcome [33]. With respect to the protective effects of APC on the kidney during sepsis, especially, the group of Gupta et al. has identified specific mechanisms of action of APC in rat models of experimentally induced septic AKI [16, 19]. In these studies, rats simultaneously received LPS and 10, 30, or 100 μg/kg APC. In the present study, the rats first received an LPS bolus, and fluid resuscitation was started 1 h later with 0, 10, or 100 μg/kg/h APC. We did not investigate the direct acting mechanisms of APC in our present study but merely focused on the acute effects of APC-supplemented fluid resuscitation in the kidneys of endotoxemic rats. Mimicking the clinical use of APC, at a rate of 24 μg/kg/h for 96 h was neither practical nor was our aim in this study.

The increase in VO_2_/T_Na+_ following LPS and fluid resuscitation without APC could either indicate less efficient oxygen use for ATP production for Na^+^ reabsorption or that oxygen is used for other purposes than ATP production such as ROS generation. In a recently published review [34], we described that ischemia/reperfusion injury, also arises during hypotensive (septic) shock and resuscitation, is associated with intrarenal microcirculatory dysfunction caused by an imbalance between vasoconstrictors and vasodilators, endothelial damage and endothelium-leukocyte interactions [35–37], oxidative stress [38, 39], and oxygen handling [40–42]. Alterations in oxygen transport pathways can result in cellular hypoxia and/or dysoxia [33]. This condition is associated with mitochondrial failure and/or activation of alternative pathways for oxygen consumption [34, 43]. This could explain the observed rise in VO_2_/T_Na+_ here.

Another explanation for the observed increase of VO_2_/T_Na+_ is the back leak phenomenon. Renal *I*/*R* injury is shown to cause derangements of the tubule cell cytoskeleton, altered integrity of tight junctions between cells and loss of epithelial polarity, ultimately providing a pathway for back leak of filtrate. These impairments are suggested to cause uncoupling of renal sodium transport and oxygen consumption, leading to inefficient sodium reabsorption. In this view, the above described results of this study might also be explained by prevention of *I*/*R*-induced renal cell injury in APC-treated rats and protective effects of APC on cellular integrity and tight junction structure [44, 45].

There is some evidence that traditional HES solutions can impair renal function and should be used with caution in patients with renal insufficiency [46]. In contrast, the latest generation of HES with low molecular weight and low degree of substitution (such as HES 130/0.4) is suggested to have minimal influence on renal function and coagulation. In 2006, Johannes et al., using phosphorescence lifetime technique, studied the influence of fluid resuscitation and fluid of choice on renal microvascular oxygenation in a similar model of endotoxemia [47]. HES 130/0.4 had least influence on renal VO_2_ and restored renal function. In this view, we have decided to choose HES 130/0.4 for fluid resuscitation during our study.

We are aware that our study suffers from some limitations. First, no markers of systemic or renal inflammation or coagulation disorders or oxidative stress were measured. Instead, we merely focused on the acute physiological effects of APC-supplemented fluid resuscitation on renal oxygenation, oxygen handling, and function. Second, endotoxemic models may not reflect all the situations encountered in human sepsis and may lack relevance in gram-positive sepsis. However, it is a reproducible model of acute inflammation that involves similar pathways and thus allows us to study the pathophysiology and potential treatment of endotoxemia-induced AKI. Extrapolation of this model to clinical scenarios in terms of treatment strategies should be made with utmost caution. Instead, our study should be regarded as adding to our understanding of the factors contributing to renal microcirculatory failure and potential treatment strategies. Third, the present study only allows assessment of the acute effects of LPS, fluid resuscitation, and APC supplementation in this short-term rat model. APC was infused for 2 h, and the experiments only lasted 3 h in total which may be insufficient to see any hemodynamic effect. Fourth, the only drug specifically approved for sepsis (recombinant human APC) has been withdrawn from the market. However, investigating its effects in experimental studies is still very relevant as it potentially provides new insights into the development and treatment of septic AKI.

## Conclusions

In conclusion, our data suggest that renal sodium reabsorption is closely correlated to renal microvascular oxygenation during experimentally induced endotoxemia. APC supplementation to standard resuscitation protocol partially protected both renal parameters. The specific mechanisms responsible for these protective effects of APC warrant further study.

## Authors’ original submitted files for images

Below are the links to the authors’ original submitted files for images. Authors’ original file for figure 1 Authors’ original file for figure 2
