# Supplementary figures and images for: Activated protein C ameliorates impaired renal microvascular oxygenation and sodium reabsorption in endotoxemic rats
Source: Intensive Care Med Exp. 2013 Oct 29;1:5. doi: 10.1186/2197-425X-1-5 (PMC4796218; doi:10.1186/2197-425X-1-5)

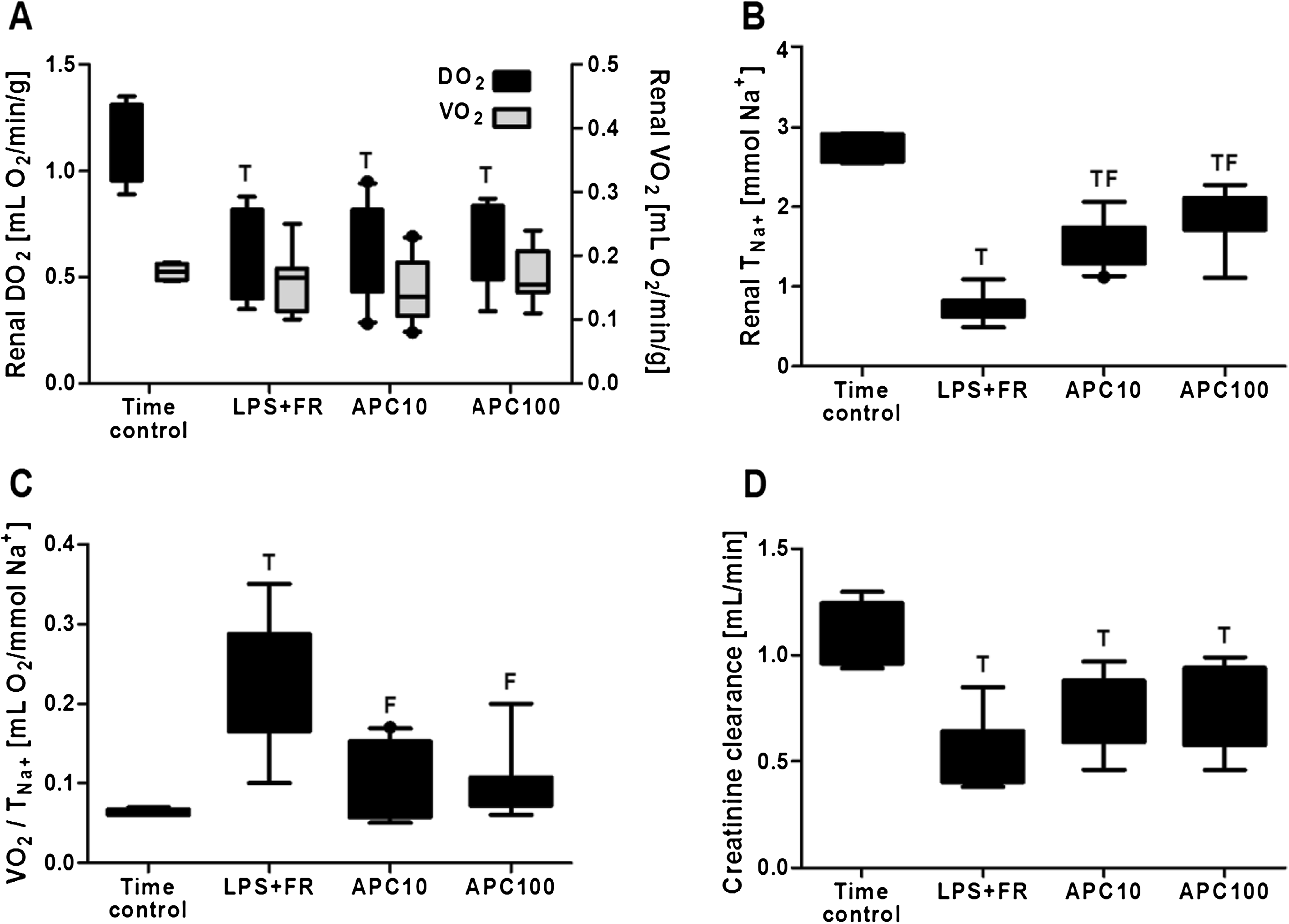

Supplement: Supplementary file 1 — Authors’ original file for figure 1 [file 40635_2013_24_MOESM1_ESM.tiff]

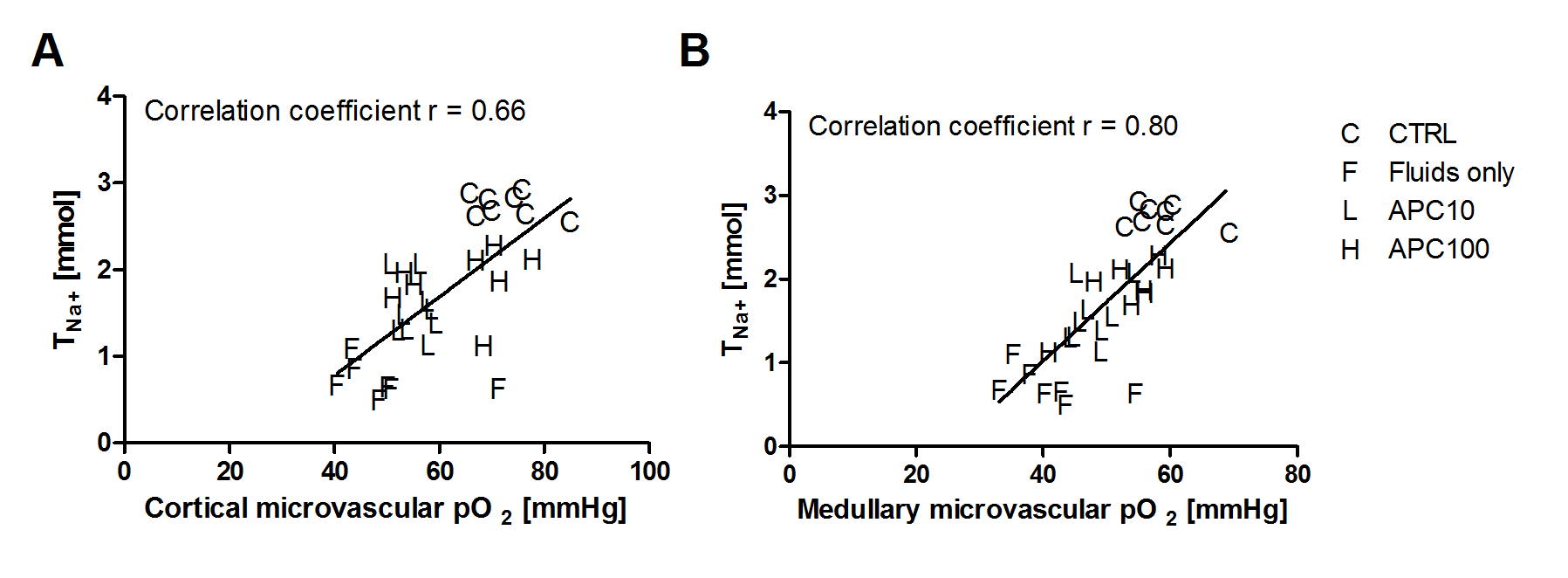

Supplement: Supplementary file 2 — Authors’ original file for figure 2 [file 40635_2013_24_MOESM2_ESM.jpeg]
